# Magnetically Recoverable Catalysts: Beyond Magnetic Separation

**DOI:** 10.3389/fchem.2018.00298

**Published:** 2018-07-18

**Authors:** Zinaida B. Shifrina, Lyudmila M. Bronstein

**Affiliations:** ^1^A.N. Nesmeyanov Institute of Organoelement Compounds, Russian Academy of Sciences, Moscow, Russia; ^2^Department of Chemistry, Indiana University, Bloomington, IN, United States; ^3^Department of Physics, Faculty of Science, King Abdulaziz University, Jeddah, Saudi Arabia

**Keywords:** magnetically recoverable catalysts, iron oxide influence, catalytic species distribution, graphene derivative, side reactions

## Abstract

Here, we discuss several important aspects of magnetically recoverable catalysts which can be realized when magnetic oxide nanoparticles are exposed to catalytic species and catalytic reaction media. In such conditions magnetic oxides can enhance performance of catalytic nanoparticles due to (i) electronic effects, (ii) catalyzing reactions which are beneficial for the final reaction outcome, or (iii) providing a capacity to dilute catalytic metal oxide species, leading to an increase of oxygen vacancies. However, this approach should be used when the magnetic oxides are stable in reaction conditions and do not promote side reactions. Incorporation of another active component, i.e., a graphene derivative, in the magnetically recoverable catalyst constitutes a smart design of a catalytic system due to synergy of its components, further enhancing catalytic properties.

## Introduction

Magnetically recoverable catalysts received considerable attention in the last decade due to possibility of magnetic separation, allowing for easy recovery with a minimal catalyst loss. In addition, it leads to conservation of energy and of a rare metal catalyst and results in cheaper target products. Several excellent reviews have been published on magnetically recoverable catalysts (Shylesh et al., 2010; Polshettiwar et al., 2011; Hudson et al., 2014; Kainz and Reiser, 2014; Rossi et al., 2014; Wang and Astruc, 2014; Sharma et al., 2016; Kang et al., 2017). In the majority of cases, magnetic nanoparticles (NPs) are isolated from the catalytic centers (immobilized catalytic complexes or catalytic NPs) using carbon (graphene) or silica coating. Coating of magnetic NPs with carbon or silica layers allows one to accomplish two goals: (i) to isolate NPs to prevent oxidation of a magnetic metal (it is imperative for Co or Fe) and/or (ii) to impart functional groups on the magnetic NP surface which is facilitated via sp^2^ carbon of graphene or silanol groups of silica. In the former case, unprotected metal nanoparticles require a strong protective shell because their high sensitivity to air makes them pyrophoric. The carbon shells provide the best protection due to their superior chemical and thermal stabilities, while silica shells do not fully block the oxygen diffusion (Schaetz et al., 2010b). The silica shells are more applicable for iron oxide or ferrite NPs. They protect magnetic cores more efficiently than, for example, long-chain alkyl surfactants (Sun and Zeng, 2002) or polymers (Caruso, 2001; Pyun, 2007) and can be formed by sol-gel method using silanes (Lu et al., 2002).

In order to implement functional groups on the surface of carbon coated magnetic NPs, diazonium chemistry was utilized (Grass et al., 2007). This approach can be modified by introducing azide groups that allow the covalent attachment of acetylene terminated molecules via Cu(I)-catalyzed “click” reaction (Schaetz et al., 2008, 2010a). Additionally, noncovalent functionalization via the carbon layer can be achieved by π-π stacking interactions (Wittmann et al., 2010), allowing, for example, the thermally reversible attachment of a pyrene-terminated Pd-containing N-heterocyclic carbene (NHC) ligand. It is worth noting that in all cases, the effective loading with functional molecules was limited to a moderate level of 0.1–0.2 mmol/g.

In the case of silica shells, silanol groups on the surface allow simple surface functionalization with various silanes and silane-modified molecules such as polymers, dendrimers, chelating ligands, catalysts, etc (Wang et al., 2013; Costa et al., 2014).

In our work, magnetically recoverable catalysts are based on superparamagnetic iron oxide NPs stabilized by dendron(dendrimer)/polymer molecules or formed in the pores of mesoporous solids. No solid shells exist around magnetic NPs which would isolate an iron oxide surface. Because magnetic iron oxides (even magnetite) are quite stable toward oxidation, exposure of the catalysts containing such NPs to air is not detrimental for their magnetic properties, ensuring the reliability of their magnetic response. In the absence of an isolating shell, the catalytic metal NPs can be in a direct contact with magnetic NPs, whose surface is also exposed to a catalytic reaction mixture. Due to these conditions, we observed a number of interesting phenomena which demonstrate that the direct exposure of magnetic NPs can be beneficial for the catalyst development and the catalytic properties.

## Iron oxide can enhance catalytic activity

**Figure 1:**
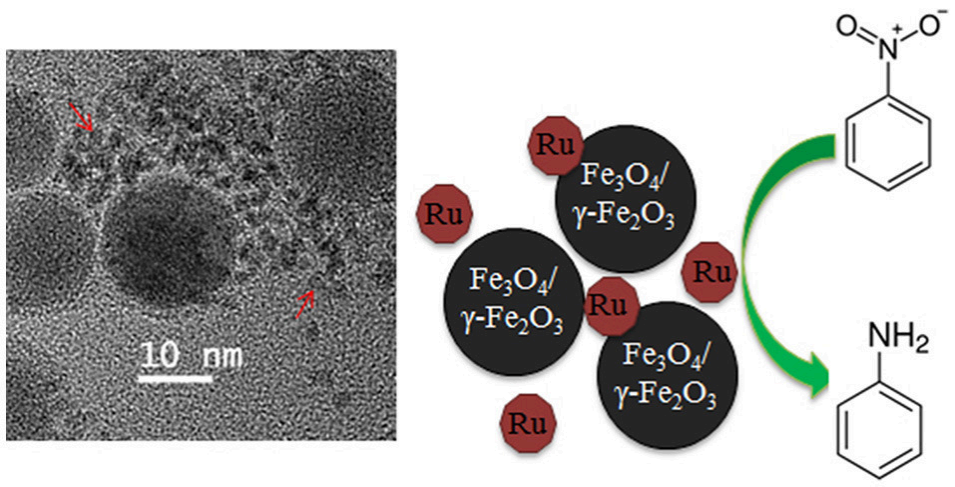
TEM image **(Left)** and schematic representation of the catalyst catalyzing hydrogenation of nitrobenzene to aniline **(Right)**. Red arrows indicate Ru NPs (Easterday et al., 2015). It is being reproduced with the permission of the copyright holder [Royal Society of Chemistry].

The influence of iron oxide NPs on catalytic hydrogenation of various substrates was demonstrated on several occasions for Pt (Gumina et al., 2013), Au (Milone et al., 2007), Pd (Easterday et al., 2014), and Ru (Easterday et al., 2015) NPs. For Pd and Ru NPs (Easterday et al., 2014, 2015), this effect is especially straightforward because the catalysts were prepared by direct combination of iron oxide NPs with Ru or Pd NPs formed *in situ* in the presence of 1,2-hexadecane diol, oleylamine, and oleic acid. Moreover, the desired effect, i.e., a significant increase of activity and selectivity in selective hydrogenation, was observed in the conditions which promoted partial aggregation of magnetic and catalytic NPs (due to polarization forces), allowing for magnetic separation and close proximity of both types of particles (Figure 1). This approach also allowed for a direct comparison with Pd and Ru NPs of the same sizes (synthesized in the analogous conditions but without iron oxide NPs), whose activity and selectivity were significantly lower than those for magnetically recoverable counterparts. Even when Pd (Ru) NPs are not attached to the iron oxide NPs, their close proximity can result in collisions, leading to interactions and electron transfer between the iron oxide NP surface and the Pd (Ru) surface which is a common phenomenon in the support-metal interactions. This results in the activation of the functional group facilitating hydrogenation (Milone et al., 2007; Cardenas-Lizana et al., 2011; Easterday et al., 2014, 2015).

## Iron oxide can change the catalytic reaction pathway

Iron oxide can change a reaction pathway due to catalyzing the process which completely changes the reaction outcome. Below we are discussing two cases, where the change of the reaction pathway was clearly documented.

Ethylene glycol (EG) and propylene glycol (PG) are known to be key precursors for pharmaceuticals, liquid fuels, emulsifiers, and surfactants (Harlin, 2011; Yue et al., 2012). One of the environmentally friendly ways of their syntheses is cellulose (a major component of biomass) catalytic hydrogenolysis in water. This reaction can be carried out as a *one-pot* process in subcritical water with various heterogeneous catalysts (Verendel et al., 2011). Ru-containing catalysts are considered the best, but produce mainly sorbitol and mannitol, while EG and PG are obtained in small amounts (Dhepe and Fukuoka, 2007; Luo et al., 2007; Kobayashi et al., 2011; Manaenkov et al., 2014). Recently we developed magnetically recoverable catalysts based on mesoporous magnetic silica (Fe_3_O_4_-SiO_2_) and Ru NPs (Manaenkov et al., 2016). In optimized reaction conditions in subcritical water, the Ru-Fe_3_O_4_-SiO_2_ catalyst allowed for the highest selectivities to EG (19%) and PG (20%) with trace amounts of sorbitol and some other compounds.

It is well documented that cellulose hydrogenolysis is a multistep process. The first step occurs in subcritical water due to formation of hydroxonium protons (Luo et al., 2007). This leads to the formation of glucose, which easily caramelizes in the absence of hydrogenation catalysts. If suitable Ru-containing catalysts are present, sorbitol is formed (Manaenkov et al., 2014). It is noteworthy that at the beginning of the reaction with Ru-Fe_3_O_4_-SiO_2_ sorbitol is also formed but it is later consumed to produce EG and PG, revealing that Fe_3_O_4_ promotes these transformations, i.e., hydrogenolysis.

To corroborate this hypothesis, we synthesized the Ru-SiO_2_ catalyst using the same mesoporous silica as precursor and the same procedure as that for Ru-Fe_3_O_4_-SiO_2_ but without Fe_3_O_4_ NP formation. The Ru-SiO_2_ catalyst allowed formation of sorbitol and mannitol (with the total selectivity of 6.5%) along with other polyols, showing that without Fe_3_O_4_ NPs hydrogenolysis barely takes place. In the case of Ru-Fe_3_O_4_-SiO_2_, these polyols are nearly absent due to efficient hydrogenolysis. Thus promoting hydrogenolysis, Fe_3_O_4_ NPs change the reaction pathway and as consequence, different reaction products (EG and PG) can be targeted.

The other example of the reaction pathway change was observed for magnetic zeolites (Mann et al., 2016). Zeolite ZSM-5 is a well-known catalyst of methanol-to-hydrocarbon (MTH) and methanol-to-gasoline (MTG) transformations (Olsbye et al., 2012). In our studies we compared the methanol conversion rates and the yields of different fractions of hydrocarbons in MTH in the presence of ZSM-5 and Fe_3_O_4_-ZSM-5, both prepared from the same mesoporous SiO_2_ precursor. While the methanol conversion rate increased by only 15% for magnetic zeolite vs. regular zeolite, the yield of hydrocarbons increased by a factor of 2.7 for Fe_3_O_4_-ZSM-5. Moreover, the yields of important hydrocarbons such C_5_-C_8_ (for synthesis of value-added chemicals) and C_9_-C_11_ (gasoline fraction) increased by more than 300 and 130%, respectively (Mann et al., 2016). Such an increase in the product yield could not be attributed to merely increased catalyst activity (15% increase). This phenomenon was puzzling until we considered the possibility of a different reaction pathway. Recently, it was reported that formaldehyde can be formed as an intermediate in the MTH reaction and it may participate in Formose-type reactions leading to carbon–carbon formation and chain growth (Sun et al., 2014). At the same time, iron-containing compounds were shown to catalyze the formaldehyde synthesis from methanol (Bowker et al., 2002; Thivasasith et al., 2015). Thus, the Fe_3_O_4_-ZSM-5 catalysts most likely allow for a higher formaldehyde yield, therefore promoting the chain growth and formation of long hydrocarbons.

## Iron oxide can become a reservoir for catalytic species

A methanol synthesis from syngas is an important sustainable process (Waugh, 2012) which is closely associated with biomass or biooil conversion to syngas (Wang et al., 2007; Seyedzadeh Khanshan and West, 2016) and syntheses of value-added chemicals such as hydrocarbons or fuels obtained from methanol. ZnO and mixed zinc containing oxides were reported to show promising catalytic properties in transformation of syngas to methanol due to oxygen vacancies in these metal oxides (Kurtz et al., 2005; Polarz et al., 2006; Strunk et al., 2009). Recently, we reported syntheses of Zn-containing magnetic oxides, prepared by thermal decomposition of Zn(acac)_2_ in the reaction solution of preformed magnetite nanoparticles (NPs) stabilized by polyphenylquinoxaline (PPQ) (Baird et al., 2016). While magnetite is not a catalyst in this reaction, the iron oxide species could behave as a dopant increasing the ZnO oxygen vacancies. However, no ZnO phase was detected in Zn-containing magnetic oxides, although the activities of these catalysts in the methanol synthesis were much higher than those of conventional catalysts. To further enhance the catalytic activity, we studied the influence of such doping metals as Ni, Co, and Cr on the structure of Zn-containing magnetic oxides and their catalytic properties in the methanol synthesis (Baird et al., 2017). Two thermally stable capping polymers have been explored: (i) linear PPQ (Singh et al., 1995; Keshtov et al., 2001) and a hyperbranched pyridylphenylene polymer (PPP) (Kuchkina et al., 2015). At low doping metal contents, a significant increase of catalytic activity has been observed (the most pronounced for hyperbranched PPP). In neither case, however, Zn or doping metal formed a separate phase and the Fe^2+^:Fe^3+^ atomic ratio of magnetite was preserved, both on the NP surface and in subsurface layers (by X-ray photoelectron spectroscopy, XPS) (Baird et al., 2017). XPS also showed that there is a gradient in Zn and doping metal contents with clear surface enrichment. These data reveal that magnetite NPs serve as reservoir for others metals, probably creating oxygen vacancies which are crucial in the syngas-to-methanol transformation.

**Figure 2:**
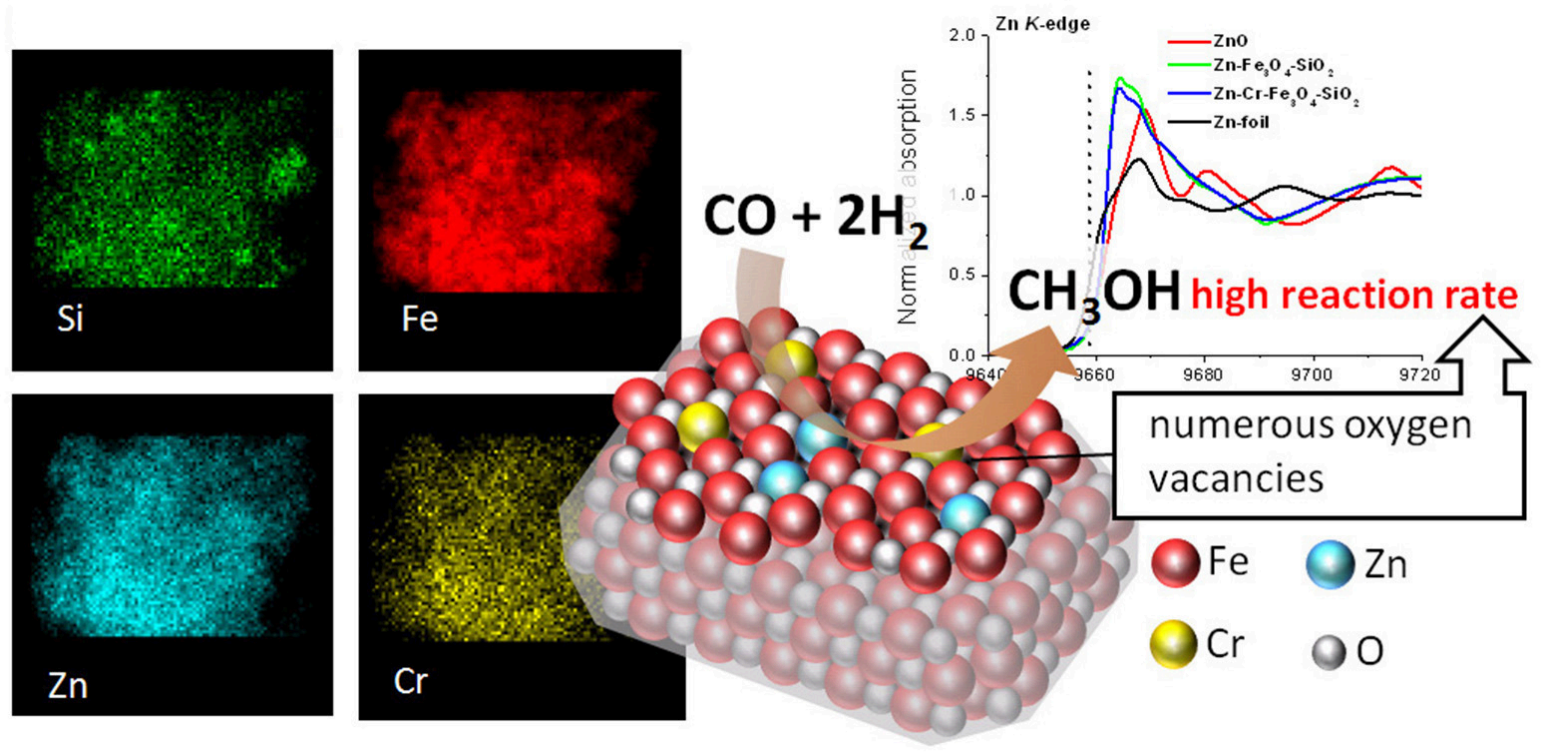
EDS elemental maps **(Left)**, normalized Zn *K*-edge XANES spectra **(Right)**, and schematic representation of Zn-Cr-Fe_3_O_4_-SiO_2_ catalyzing the methanol synthesis **(Center)** (Oracko et al., 2017). It is being reproduced with the permission of the copyright holder [American Chemical Society].

This approach was further extended to magnetically recoverable catalysts based on magnetic silica (Oracko et al., 2017). Similar to the polymer stabilized Zn-containing magnetic oxides, incorporation of Zn and doping metal species resulted in a single magnetite phase with a gradient of the Zn and the doping metal contents from the surface to subsurface layers (Figure 2). Furthermore, X-ray absorption spectroscopy (EXAFS and XANES) revealed that the catalyst structure is different from Fe_3_O_4_ or ZnO or Cr_2_O_3_. Instead, magnetic oxides demonstrated a significant shortage of oxygen atoms around Fe, Zn, and Cr, i.e., oxygen vacancies which increased from a single oxide phase (for example, Fe_3_O_4_ or ZnO) to Zn-Fe_3_O_4_-SiO_2_ and further to Zn-Cr-Fe_3_O_4_-SiO_2_, resulting in a significant increase of catalytic activities of these magnetic oxides (Oracko et al., 2017).

## Iron oxide can be detrimental to the reaction outcome

There are two major avenues for iron oxide NPs being detrimental for the catalytic reaction outcome: (i) dissolution of iron oxide NPs in an acidic medium, leading to a loss of magnetic recovery or (ii) catalyzing side reactions, which could result in undesired products. The former phenomenon was observed in hydrogenation of bio-oil into higher alcohols with Ru-containing magnetic silica containing magnetite and Ru NPs in the silica pores (Cherkasov et al., 2017). In optimal conditions at pH 3 the highest hydrogenation rate was achieved at the minimum hydrogen and energy consumption, however, a noticeable dissolution of iron oxide was observed. When pH was adjusted to 6.0, the rate of hydrogenation of furfural and phenol decreased by a factor of 2. Finally, it was determined that the pH of 4.5 allows a reasonable compromise between a hydrogenation reaction rate and the catalyst deactivation rate via dissolution.

A side reaction was found to be a problem in the furfural hydrogenation to furfuryl alcohol with magnetically recoverable catalysts containing Pd and Pt NPs stabilized by PPQ and PPP (Alibegovic et al., 2017). The control experiments carried out with magnetite NPs stabilized by these polymers showed that the furfural conversion occurs with both Fe_3_O_4_-PPQ and Fe_3_O_4_-PPP, giving 15.3 and 2.7% of *i*-propyl-furfural ether, respectively. For the optimized Pd-Fe_3_O_4_-PPQ, the selectivity to furfuryl alcohol did not exceed 88.7% due to formation of 10.3% of *i*-propyl-furfural ether (from 2-propanol solvent). On the other hand, for optimized Pd-Fe_3_O_4_-PPP, the selectivity to furfuryl alcohol was 99.4% with only 0.6% of *i*-propyl-furfural ether. Clearly, in this case, iron oxide is detrimental to the reaction outcome, but for PPP, the side reaction is minimized. This is explained by tethering of catalytic NPs by a hyperbranched polymer increasing the distance between catalytic and magnetite NPs and better protection/isolation of the iron oxide surface with a large amount of the polymer.

## Synergy of iron oxide with graphene derivative supports

In magnetically recoverable catalyst, iron oxide is often a support for catalytic NPs along with a polymer or a mesoporous solid. At the same time, magnetic nanocomposites could benefit from an additional active support component which could play an important role in a catalyst formation and in a catalytic reaction. In recent years, graphene derivatives such as graphene oxide (GO), reduced GO (RGO), N-doped graphene, etc. have been introduced as catalysts and catalyst supports (Singh et al., 2015; Das et al., 2017). The remarkable feature of graphene derivatives containing a significant fraction of sp^2^ carbon atoms is that they are capable of directing the formation of metal or metal alloy NPs along the certain crystalline planes, thus, controlling the catalyst structure (Dahal and Batzill, 2014). We have developed a novel magnetically recoverable nanocomposite based on partially reduced GO (pRGO), polyethyleneimine (PEI), magnetite and Ru NPs, which showed remarkable regio- and chemoselectivity in transfer hydrogenation of nitrobenzene to aniline using 2-propanol as a hydrogen source (unpublished). We discovered that Fe_3_O_4_ NP formation in the presence of GO and PEI results in partial reduction of GO to pRGO, whose structure directs the Ru or Ag NP formation and creates synergy between the catalyst components (Das et al., 2018). This synergy is reflected in the catalyst behavior, as pRGO allows for adsorption of nitrobenzene and intermediates, while Ru NPs catalyze hydride transfer, with magnetite NPs making the system magnetically recoverable.

## Summary and outlook

In this perspective we highlight the importance of many aspects of magnetically recoverable catalysts which are often overlooked when such catalysts are developed and studied. Careful design of magnetically recoverable catalytic systems can enhance activities of the catalysts via several effects such as the electron transfer from the magnetic NP surface, change of the reaction pathway or distribution of catalytic species within iron oxide NPs, leading to their unique properties. These effects, however, can be only observed when magnetic NPs are unprotected by any solid shells, thus, the magnetic NPs need to be stable in reaction conditions and do not catalyze any side reactions.

Despite the title of this paper (“Beyond magnetic separation”), we would like to emphasize that easy magnetic separation is still a very important attribute of magnetically recoverable catalysts. Moreover, the catalysts with high cooperative magnetic moments, can be used for magnetic catalyst fixation in continuous-flow processes (Park and Kim, 2010; Schaetz et al., 2010a; Rehm et al., 2015), which constitutes the future for commercialization of magnetically recoverable catalysts.

## Author contributions

All authors listed have made a substantial, direct and intellectual contribution to the work, and approved it for publication.

### Conflict of interest statement

The authors declare that the research was conducted in the absence of any commercial or financial relationships that could be construed as a potential conflict of interest.
